# Dietary diversity score (DDS) and odds of colorectal cancer and adenoma: a case–control study

**DOI:** 10.1017/jns.2022.30

**Published:** 2022-05-10

**Authors:** Alireza Bahrami, Pedram Shirani, Mohammadhassan Sohouli, Saeede Jaafari Nasab, Pegah Rafiee, Farah Naja, Zahra Sheikhimobarakeh, Ehsan Hejazi

**Affiliations:** 1 Department of Clinical Nutrition and Dietetics, School of Nutrition Sciences and Food Technology, Shahid Beheshti University of Medical Sciences, Tehran, Iran; 2 Department of Community Nutrition, School of Nutrition Sciences and Food Technology, Shahid Beheshti University of Medical Sciences, Tehran, Iran; 3 Department of Nutrition, School of Public Health, Iran University of Medical Sciences, Tehran, Iran; 4 Department of Nutrition and Food Science, Faculty of Agricultural and Food Sciences, American University of Beirut, Beirut, Lebanon; 5 Quality of Life Department, Breast Cancer Research Center, Motamed Cancer Institute, ACECR, Tehran, Iran

**Keywords:** Diet quality, Dietary diversity, Colorectal cancer

## Abstract

Despite mounting evidence that dietary factors might have a protective role against risk of cancer, few studies have assessed the relationship between diet diversity with colorectal cancer (CRC) and colorectal adenoma (CRA). Thus, we examined the relationship between dietary diversity score (DDS) and the odds of CRC and CRA. Overall, 129 CRC diagnosed patients, 130 CRA diagnosed cases and 240 healthy hospitalised controls were studied. DDS was calculated based on information on the usual diet that was assessed by a valid and reliable food frequency questionnaire (FFQ). Multivariate logistic regression was used to estimate the relationship between DDS and odds of colorectal cancer and adenoma. After adjusting for potential confounders, the diversity of grains is associated with the increased odds of CRC (OR_grains_: 2·96 (1·05–8·32); *P* = 0·032), while the diversity of vegetables and fruits are associated with decreased odds of CRC (OR_vegetables_: 0·31 (0·16–0·62); *P* = 0·001, OR_fruits_: 0·37 (0·23–0·61); *P* < 0·001). The diversity of vegetables, fruits and dairy are inversely associated with odds of CRA (OR_vegetables_: 0·41 (0·21–0·78); *P* = 0·007, OR_fruits_: 0·58 (0·36–0·93); *P* = 0·021, OR_dairies_: 0·56 (0·37–0·83); *P* = 0·004). Also, higher DDS was related to decreased odds of both CRC (OR: 0·41 (0·23–0·72); *P* for trend = 0·002) and CRA (OR: 0·36 (0·21–0·65); *P* for trend = 0·001). Our results indicated that higher dietary diversity and particularly a diet varied in fruits and vegetables may reduce the odds of CRC and CRA. Also, the consumption of dairy products may decrease the odds of CRC, whereas the consumption of grains may increase the odds of CRC.

## Introduction

According to GLOBOCAN 2018 data, colorectal cancer (CRC) is the third most commonly diagnosed cancer in the world^(1)^. Due to the ‘western’ way of life, the incidence of CRC is increasing especially in developing countries, so that nearly 2 million new cases are diagnosed in 2018^(2)^. In Iran, a developing country, CRC is the fourth and third most common cancer in Iranian males and females, respectively, and the incidence of this malignancy has increased in recent years^(3)^. CRC is a multifactorial disease involving several non-modifiable factors (such as genetic, advanced age, male sex and family history) and modifiable factors (such as alcohol consumption, dietary factors, smoking and obesity)^(4)^. In recent decades, epidemiological studies have investigated diet as a potential modifiable factor. Results of these studies indicated that a diet rich in fruits, vegetables and dairy products is related to a decreased risk of CRC, while higher consumption of red and processed meat and saturated fat has been associated with increased risk of CRC^(5–10)^. Furthermore, a few studies highlighted the role of the diversity in the diet and proposed that a more diverse diet could play a protective role against some types of cancers^(11–13)^. Dietary diversity score (DDS) is a useful approach to evaluate the variety of foods consumed and total diet quality^(14)^. Results from a case–control study conducted in Italy showed that total dietary diversity and diversity in vegetables were associated with decreased risk of CRC^(15)^. Another case–control study conducted in Western New York found an inverse association between total dietary diversity and colon cancer risk among men only^(16)^. In contrast, Slattery *et al.* identified no association between total and specific food group diversity and risk of colon cancer^(17)^. Due to inconsistent results and to provide more information on the role of dietary diversity on the risk of CRC and its precursors, colorectal adenoma (CRA)^(18)^, the present study aimed to examine the relationship between dietary diversity, as measured by the DDS, and the odds of CRC and CRA.

## Methods

### Participants

A hospital-based case–control study involving newly diagnosed (<3 months) patients with CRC and CRA was carried out. Cases and controls were recruited from three major general hospitals (Taleghani, Imam Hussein, Shohadaye Tajrish) in Tehran, Iran (October 2016 to May 2018). CRC and CRA cases were patients with pathologic confirmation and colonoscopy findings, aged 30–79 and had no history of cancers of other sites and previous diagnosis of CRA. Controls were assigned from patients visiting the same hospital at the same time and same setting. Controls were frequency-matched with cases by sex and age (10-year groups). The control group consisted of patients aged (30–79 years) admitted to the same hospital as cases for a wide spectrum of acute diseases that were not associated with long-term modification of the diet. Controls were hospitalised mainly because of the following conditions: traumas (25·9 %, mostly fractures and sprains), surgical conditions (20·1 %, mostly abdominal such as acute appendicitis and kidney stones), no traumatic orthopaedic conditions (4·2 %, mostly disk disorders and back pain) and miscellaneous diseases (49·8 %, including acute eye, nose, skin and throat disorders). Participation rate in the study was 92%. Overall, 129 CRC and 130 CRA cases and 240 controls participated in this study. The protocol of the present study was approved by the Ethics Committee of Shahid Beheshti University of Medical Sciences with the ethic code of IR.SBMU.NNFTRI.REC.1397.041.

### Dietary assessment

Information about usual dietary intake of the cases (1 year before diagnosis) and the controls (1 year before interview) was collected using a valid and reliable semi-quantitative food frequency questionnaire (FFQ) that consists of 148 food items with standard serving size commonly consumed in Iran^(19)^. Controls were asked how often, on average, per week or per day during the last year they had consumed these foods. Cases were asked before your disease diagnosis, how often, on average, per week or per day, they had consumed these foods. The frequency of consumption of given serving of each food item was questioned on a daily (e.g. bread), weekly (e.g. meat) or monthly (e.g. fish) basis, and data were transformed into the average daily intakes, assuming one month equal 30·5 d. The portion sizes were then converted to grams by using the household scales^(20)^. The consumption of food items in grams was then calculated by multiplying the portion size by daily intake frequency. Daily energy, macronutrients and micronutrients consumption for participants was computed by Nutritionist IV software. Since the Iranian food composition table (FCT) is not complete and comprehensive, analysis of energy and nutrients were done using the United States Department of Agriculture (USDA) food composition table.

### Assessment of other covariates

In addition to dietary intake assessment, participants were asked about the following: socio-demographic characteristics, family history of cancer, family history of CRC, smoking habit and medical information (comorbidities, use of drugs and vitamin/mineral supplements). The weight of each individual with the least amount of clothing and a sensitivity of 100 g and height without shoes with a sensitivity of 0·1 cm were measured. All participants were asked to complete a physical activity questionnaire and rate their daily activities such as walking, exercise, sleep, watching television, housework and job-related tasks. Total activity was reported for 1 d, and the metabolic equivalent of tasks (METs) were calculated based on these reports^(21)^.

### DDS assessment

To calculate the DDS, we used the method of Kant *et al.*^(14)^ that it was modified in a study in Iran^(22)^ and we used this modified version. According to this method, food items were categorised to five food groups comprising vegetables, fruits, bread and grains, meats and dairies. This classification was based on food guide pyramid^(23)^. Using the FFQ data collected in this study, the vegetables group consisted of seven subgroups: vegetables, potatoes, tomatoes, other starchy vegetables (corn, pea, eggplant, squash), legumes (peas, beans, mung beans, split peas, lentils), yellow vegetables (carrots and pumpkin) and other green vegetables (bell peppers, all kinds of cabbage, broccoli, celery, cucumbers, garlic, onion, green beans, zucchini, leeks, parsley, lettuce, radish, spinach, rhubarb, turnips). For the fruit group, fruit and fruit juice, berries and citrus were included. Grains group with seven subgroups: white bread (lavash bread), whole grain biscuits, pasta, whole grain breads (Sangak, taftoon, barbari), noodle, rice, barley. Meat group with four subgroups consisted of red meat (cattle and sheep), poultry (hen and chicken), fish and eggs. The dairy group included three subgroups including milk (low-fat and full-fat), yoghurt (low-fat and full-fat), cheese or curd. Individuals should have consumed at least half serving of any subgroups of each food group per day, to be classified as a consumer of any mentioned food group. Each food group has a maximum of 2 scores. Therefore, the minimum DDS was zero and the maximum was ten.

### Statistical analysis

Data analysis was performed by Statistical Package Software for Social Science, version 21 (SPSS Inc., Chicago, IL, USA). The normality of the data was checked using Kolmogorov–Smirnov's test. Comparisons of baseline characteristics and dietary intakes between cases and controls were done using *χ*^2^ test for categorical variables and independent sample *t*-test for continuous variables, respectively. Logistic regression was used to determine the odds ratio (OR) with a 95 % confidence interval (CI) of CRC and CRA by food group diversity and tertile of DDS. In the multivariable model, the potential confounding variables including comorbidity, cancer family history, CRC family history, common ways of cooking food, physical activity and calcium supplement were adjusted. OR and 95 % CI were reported, and *P*-values <0·05 were considered statistically significant.

## Result

The socio-demographic and lifestyle characteristics of the participants are presented in Table 1. By frequency-matched design, cases and controls had no significant difference age or sex distribution. Cancer cases were more likely to have family history of cancer among first degree relatives and significantly lower physical activity than controls. While adenoma cases were more likely to have at least one comorbidity and higher intake of daily calcium supplement intake compared to controls. Also, there is a significant difference in common ways of cooking food between adenomas and controls group, patients with adenomas commonly consume grilled and combined foods, whereas controls commonly consume boiled foods. There was no statistically significant difference among the groups concerning other variables. The distribution of dietary intakes of participants across the tertiles of DDS is shown in Table 2. According to this table, subjects in lower DDS tertiles had significantly lower energy and nutrients intake. Table 3 shows the ORs and 95 % CIs for CRC and CRA risk by food groups diversity. After adjusting for potential confounders, the diversity of grains is associated with the increased odds of CRC (OR_grains_: 2·96 (1·05–8·32); *P* = 0·03), while the diversity of vegetables and fruits are associated with decreased odds of CRC (OR_vegetables_: 0·31 (0·16–0·62); *P* = 0·001, OR_fruits_: 0·37 (0·23–0·61); *P* < 0·0001). In relation to CRA, the diversity of vegetables, fruits and dairies are inversely associated with odds of CRA (OR_vegetables_: 0·41 (0·21–0·78); *P* = 0·007, OR_fruits_: 0·58 (0·36–0·93); *P* = 0·02, OR_dairies_: 0·56 (0·37–0·83); *P* = 0·004). Table 4 presents the ORs and 95 % CIs for CRC and CRA odds according to tertiles of DDS. After controlling for confounding factors, higher DDS was related to decreased odds of both CRC (OR: 0·41 (0·23–0·72); *P* for trend = 0·002) and CRA (OR: 0·36 (0·21–0·65); *P* for trend = 0·001).

**Table 1.** The main characteristics of the participant's study

Variables	Controls (*n* 240)	Cancer cases (*n* 129)	Adenoma cases (*n* 130)	*P*-value*	*P*-value**
Age (mean ± sd)	55·08 ± 9·45	56·6 ± 11·5	56·46 ± 10·01	^a^	^a^
Sex (male) *n* (%)	133 (55·4)	66 (51·2)	59 (45·4)	^a^	^a^
BMI (mean ± sd)	26·93 ± 3·99	26·68 ± 5·49	26·72 ± 3·81	0·62	0·63
Educational level *n* (%)				0·71	0·48
Illiterate	31 (13)	18 (14·1)	14 (10·9)		
Low education	172 (72)	94 (73·4)	90 (70·3)		
High education	34 (14·2)	16 (12·5)	24 (18·8)		
Smoking (yes) *n* (%)	42 (17·5)	26 (20·2)	27 (20·8)	0·53	0·11
Comorbidity (yes) *n* (%)	41 (17·1)	21 (16·3)	40 (30·8)	0·84	**0**·**002**
Family history of cancer in first degree (yes) *n* (%)	89 (32·9)	66 (51·2)	48 (36·9)	**0**·**001**	0·43
Colorectal cancer family history in first degree (yes) *n* (%)	18 (7·5)	10 (7·8)	17 (13·1)	0·15	0·08
Common ways of cooking food *n* (%)				0·25	**0**·**01**
Fried	55 (22·9)	40 (31)	18 (13·8)		
Boiled	81 (33·8)	41 (31·8)	34 (26·2)		
Grilled	5 (2·1)	0 (0)	4 (3·1)		
Steam cook	3 (1·3)	2 (1·6)	0 (0)		
Combined	96(40)	46 (35·7)	74 (56·9)		
Physical activity (mean ± sd) (met/h/d)	40·06 ± 9·87	36·61 ± 15·11	38·54 ± 9·39	**0**·**008**	0·14
Calcium supplement (yes) *n* (%)	35 (14·6)	28 (21·7)	32 (24·6)	0·08	**0**·**01**
Energy intake (mean ± sd)	2367·42 ± 673·1	2272·14 ± 574·02	2303·6 ± 669·9	0·17	0·38

Bold *p*-values are statistically significant.

MET, Metabolic equivalent.

a Matched variables of the study.

* *P*-value between cancers and controls.

** *P*-value between adenomas and controls independent sample *t*-test was used for continuous variables and *χ*^2^ was used for categorical variables.

**Table 2.** Participants’ dietary intakes according to DDS tertile

Dietary intake^a^	DDS tertile categories			
	T1 (<3·42)	T2 (3·42–5·85)	T3 (>5·85)	*P*-value^b^
Total energy (kcal/d)	2050·98 ± 661·58	2364·18 ± 521·93	2577·37 ± 619·02	<0·0001
Carbohydrate (g/d)	175·90 ± 71·33	262·43 ± 91·23	308·02 ± 89·49	<0·0001
Protein (g/d)	44·19 ± 16·97	67·45 ± 20·64	85·64 ± 21·33	<0·0001
Total fat (g/d)	81·96 ± 41·77	102·31 ± 34·58	112·51 ± 31·45	<0·0001
Cholesterol (mg/d)	155·61 ± 111·46	210·12 ± 108·71	275·13 ± 118·33	<0·0001
Total fibre (g/d)	20·81 ± 17·74	30·49 ± 17·65	35·74 ± 16·75	<0·0001
Calcium (mg/d)	477·41 ± 211·23	695·41 ± 222·18	978·87 ± 268·38	<0·0001
Vitamin C (mg/d)	45·11 ± 26·76	91·72 ± 49·23	127·81 ± 54·21	<0·0001

a Data are expressed as mean ± sd.

b ANOVA test was used.

**Table 3.** Odds ratio (OR) and 95 % confidence interval (95 % CI) of colorectal cancer and adenoma in relation to food groups diversity

Food group diversity	Colorectal Cancer OR (95 % CI)^a^	Colorectal Adenoma OR (95 % CI)^a^
Grains diversity score	2·96 (1·05–8·32)	0·71 (0·25–1·93)
Vegetable diversity score	0·31 (0·16–0·62)	0·41 (0·21–0·78)
Fruit diversity score	0·37 (0·23–0·61)	0·58 (0·36–0·93)
Dairy diversity score	0·72 (0·49–1·06)	0·56 (0·37–0·83)
Meat diversity score	1·29 (0·73–2·27)	1·44 (0·84–2·46)

Logistic regression was performed to obtain the odds ratio (95 % CI) of colorectal cancer and adenoma.

a Adjusted for age, cancer family history, CRC family history, common ways of cooking food, physical activity and calcium supplement use.

**Table 4.** Odds ratio (OR) and 95 % confidence interval (95 % CI) of colorectal cancer and adenoma, according to tertile of total dietary diversity score (DDS)

Total dietary diversity score	Case/Control	Colorectal cancer OR (95 % CI)^a^	Colorectal adenoma OR (95 % CI)^a^
Tertile 1 (<3·42)	8/13	1·00 (Ref)	1·00 (Ref)
Tertile 2 (3·42–5·85)	62/81	0·47 (0·27–0·83)	0·79 (0·46–1·53)
Tertile 3 (>5·85)	59/146	0·41 (0·23–0·72)	0·36 (0·21–0·65)
*P* for trend		0·002	0·001

Logistic regression was performed to obtain the odds ratio (95 % CI) of colorectal cancer and adenoma.

a Adjusted for age, cancer family history, CRC family history, common ways of cooking food, physical activity and calcium supplement use.

## Discussion

In the present study, we found an inverse relationship between DDS and the odds of CRC and CRA. Additionally, the diversity of fruits and vegetables was related to decreased odds of CRC, while the diversity of grains was found to be associated with an increased odd of CRC. This may be because in the present study, cancer patients consumed lower amounts of whole grains and higher amounts of refined grains^(24)^. Diet diversity scores for vegetables, fruits and dairies have also been linked to a decreased odd of CRA. Overall, according to our findings, an inverse association between DDS and CRC could be attributed to higher consumption of the healthier food groups associated with higher DDS such as vegetables, fruits and dairies groups. Diet diversity is a combination of different food items that contain different food micro nutrients and compounds; for example, carotenoids, vitamin C and E, flavonoids and phytosterols are all known to have strong antioxidant and anti-carcinogenic properties^(25)^.

Our findings in relation to fruits were generally consistent with previous studies^(16)^. A population-based case–control study of Western New York indicated a strong inverse association between consumption of fruits and CRC^(16)^. Another case–control study found that moderate consumption of fruits has a protective effect on CRC. Fruits are a rich source of vitamin C, mostly known for its antioxidant property. Vitamin C can inhibit nitrosamine formation *in vivo*, inhibits mutagenesis and carcinogenesis *in vitro*, and lowers tumour cell growth and carcinogen-induced DNA damage^(26)^. Additionally, other components of fruit include phytochemicals, dietary fibres and carotenoids, which are also known to be antioxidants, and have been shown to have anti-carcinogenic effects on another cancer^(27)^. Consistent with most previous literature, we observed an inverse association between consumption of vegetables and CRC^(15–17,28)^. In a recent meta-analysis of cohort studies, a significant decreased risk of the disease was observed with overall consumption of vegetables^(8)^. Vegetables are rich sources of isothiocyanates and also other compounds such as phytochemicals, dietary fibres and carotenoids. They are known to be chemopreventive agents with anticancer mechanisms, including stimulation of apoptosis, induction of carcinogen detoxification and arrest of cell cycle progression^(29)^.

Several studies have examined the relationship between the consumption of dairy products and the risk of CRC^(15,17)^, but results of these studies were generally inconsistent. Our results are consistent with a recently published meta-analysis of nineteen studies that examined milk consumption and CRC. In this meta-analysis, the authors found a decreased risk of CRC in the highest category compared with the lowest category of milk consumption^(9)^. Several mechanisms could explain the inverse association observed between dairy intake and risk of CRC. Dairy products are the main source of dietary calcium and it has been reported that the reduced risk of cancer is partly associated with the consumption of calcium. Another possible mechanism is that fermented dairy products contain lactic acid bacteria, which have been shown to suppress CRC carcinogenesis^(30)^.

According to our findings, a positive association between grains and CRC was observed. This association might be due to lower consumption of whole grains and higher consumption of refined grains (data not shown). In a recent meta-analysis including eight studies, a decreased CRC risk was observed with high consumption of whole grains^(10)^. Whole grains contain antioxidants that may protect colonic epithelial cells against oxidative damage, and fermentable carbohydrates, which, if undigested, are fermented by short chain fatty acid-producing gut microbial species that compete against Gram-negative proinflammatory bacteria^(31)^. In contrast, refined grains, although enriched with B vitamins, contain minimal dietary fibre, and may have less impact on gut microbial diversity and abundance of beneficial species in comparison to whole grains^(32)^.

The strengths of the present study include a high response rate of participants and using use of a validated semi-quantitative FFQ which is unique to the Iranian population. Also, to reduce interviewer bias, interviewers were rigorously trained in their interview skills. The present study has some of the potential limitations of hospital-based case–control studies. As with other studies, there is a possibility of measurement error in this study. Also, it is difficult to avoid selection and recall bias. However, use of new patient cases, using hospital controls and administering FFQs by trained interviewers in a hospital setting reduced these biases.

## Conclusion

To our knowledge, this is the first study to investigate the relationship between DDS and CRC and CRA in detail in the population of Iran. Our results indicated that higher dietary diversity and particularly a diet varied in fruits and vegetables may reduce the risk of CRC and CRA. Also, the consumption of dairy products may decrease the risk of CRC, whereas the consumption of grains (low intake of whole grains *v*. high intake of refined grains) may increase the risk of CRC. These findings add to epidemiologic evidence that supports the dietary guidelines for a more diverse diet. Further studies are needed to investigate the association of DDS and the risk of CRC using longitudinal designs.
